# Sociodemographic Determinants of Malaria among Under-Five Children in Ghana

**DOI:** 10.1155/2014/304361

**Published:** 2014-12-14

**Authors:** Samuel Harrenson Nyarko, Anastasia Cobblah

**Affiliations:** ^1^Department of Population and Behavioural Sciences, School of Public Health, University of Health and Allied Sciences, Hohoe, Ghana; ^2^Department of Population and Health, University of Cape Coast, Cape Coast, Ghana

## Abstract

*Background*. Malaria is an entrenched global health challenge particularly in the sub-Saharan African countries. However, in Ghana, little is known about the determinants of malaria prevalence among under-five children. As such, this study sought to examine the sociodemographic factors that determine malaria among under-five children in Ghana. *Methods*. This paper used secondary data drawn from the 2008 Ghana Demographic and Health Survey. Bivariate analysis and complementary log-log regression models were used to examine the determinants of malaria prevalence among under-five children in Ghana for the study period. *Results*. The results therefore revealed that region of residence, age of child, and ownership of mosquito net were the key predictors of malaria cases among under-five children in Ghana for the five-year period preceding the survey. *Conclusion*. It is therefore imperative that special education on prevention of malaria should be intensified by the National Malaria Control Programme in all the regions in order to reduce malaria prevalence particularly among under-five children in Ghana.

## 1. Introduction 

Malaria is a major public health concern in the world and more especially among pregnant women and children because of their vulnerability. As such, malaria has been described as an entrenched global health challenge [1]. The disease is endemic in over hundred countries in the world. Approximately half of the world's population is at risk of malaria and nearly one million people die of the disease each year [2, 3]. Most malaria cases and deaths occur in sub-Saharan Africa. However, Asia, Latin America, the Middle East, and parts of Europe are also affected to a lesser extent. Hence, malaria affects children of all regions both poor and rich [2].

Globally, there were 216 million cases of malaria in 106 endemic countries and territories in the world in 2010, out of which 86 percent of the victims were children under five years of age. The world malaria report of 2011 shows an estimated 655,000 malaria deaths in the world, majority of which were under-five children from Africa [2]. Thus, it remains a leading cause of death in children under five years [4]. The World Health Organisation and United Nations Children's Education Fund (UNICEF) also indicate in the African Malaria Report that an outrageously higher number of over 3,000 children die from malaria in Africa daily with a child dying every 30 seconds [5].

Malaria is also a major threat to public health in Ghana and the burden of the disease is a challenge to child survival. It is also the leading cause of morbidity and mortality among children under five years in the country [6]. Besides, it has been identified as the leading cause of absenteeism in school age children in Ghana [7]. Out of the 3.5 million cases of suspected malaria cases that were reported to the public health facilities in Ghana, 20,000 were children under the age of five. The disease thus accounts for 61 percent of hospital admissions of under-five children and 25 percent of under-five mortality [8].

A number of studies have been conducted on malaria among under-five children and had attributed the disease to nonuse of insecticide treated nets (ITNs) by care givers [9]. Ahorlu et al. [10] among other studies have also conducted studies on malaria-related beliefs and behaviours, treatment, prevention, and control in Southern Ghana. However, little is known about sociodemographic determinants of malaria prevalence among under-five children in Ghana. Consequently, this paper sought to examine the sociodemographic factors that determine malaria cases among under-five children in Ghana.

## 2. Materials and Methods

This paper used secondary data drawn from the 2008 Ghana Demographic and Health Survey (GDHS) children's data file. The GDHS is a nationally representative survey for all women aged 15–49 and all men aged 15–59. The sample points cover households sampled from all over the country. The survey collected detailed reliable information on fertility levels, marriage, sexual activities, malaria, fertility preferences, awareness and use of family planning methods, awareness and behaviour regarding AIDS and other sexually transmitted infections [11]. In acquiring the dataset, a request was made from Measure DHS website on April 1, 2011, and an approval was then granted to download the data.

The GDHS was carried out by the Ghana Statistical Service (GSS), the Ghana Health Service (GHS), and ICF Macro International. The survey was conducted between September 8 and November 25, 2008, on a nationally representative sample of 12,323 households [11]. Out of a total number of 4,916 women who participated in the survey, 2,725 women who had children within the five-year period preceding the survey were selected for the study. The survey obtained information on women's exposure to malaria during their most recent pregnancy in the five years preceding the survey and the treatment for malaria. They were also asked if any of their children born in the five years preceding the survey had malaria, whether these children were treated for malaria, and about the type of treatment they received.

The statistical package STATA (version 11) was used to process the data. Two main levels of analysis were done. Firstly, bivariate analysis was done to determine proportions of malaria cases within the various selected variables. Hence, results were presented in frequencies and proportions. Secondly, complementary log-log regression model was applied to study the factors that determine malaria cases among under-five children. It was also used to predict the odds of malaria cases among the under-five children; hence, results had been presented in odds ratios (OR). This was done in four chronological models by adding factors in stages in order to identify consistent and robust predictors or determinants of malaria. Complementary log-log regression model was used because of asymmetric distribution of data in terms of malaria cases [12] and the fact that the outcome variable has a binary response denoting whether or not a child had malaria. Thus, with the outcome variable being malaria, children who had no malaria were coded “0” while those who had malaria were coded “1.” Some of the independent variables were recoded to suit the study while some were used as they are found in the original dataset.

## 3. Results 

### 3.1. Sociodemographic Characteristics and Proportion of Children with Malaria

Table 1 shows the results of proportion of children who had malaria by their sociodemographic characteristics. The highest prevalence of malaria of 26 percent was found among children aged 12–23 months while 24 percent was found among children aged 24–35 months. Malaria was less common among children aged less than 12 months (12 percent) and those aged 48–59 months (16 percent). About 21 percent of the males were found to have had malaria compared to 19 percent for their female counterparts. Highest proportion of malaria was also observed among children whose mothers were aged 40–49 years (23.8%) while the least reported malaria cases in children were among mothers aged 15–19 (18.2%). Variation in the rural-urban dichotomy showed that the proportion of children with malaria from the rural areas was 21 percent and that for children from the urban areas was 19 percent.

In terms of regional variations, the highest proportion of malaria was found among children from the Brong Ahafo Region (27%) while the Ashanti Region was the second highest (25%) and the Western Region had the least proportion of malaria with about 11 percent. For religious affiliation, the highest proportion of malaria was found among children whose mothers had no religion (26.7%) while children of mothers who professed to be traditionalists or spiritualists had the least proportion of malaria of about 12 percent. Concerning ethnicity, the Mole-Dagbani mothers and those from other ethnic groups reported the highest proportion of malaria among under-five children (21.9% and 21.7%, resp.) while the lowest was reported by Ewe mothers and Ga-Dangme mothers (15.2% and 15.6%, resp.). The results from Table 1 further show that the highest proportion of children with malaria was among children of divorced mothers (29.7%) while the lowest proportion was reported among children of mothers who were never married (17.3%).

On mother's education, the highest proportion of malaria among under-five children was reported among children whose mothers had primary school education (22.7%) while mothers with higher education reported the lowest malaria cases among children (17.4%). The results from Table 1 also show that the highest proportion of children who contracted malaria was among mothers from poor households (16.9%) and the lowest was reported among rich households (16.9%). The total proportion of malaria among under-five children in Ghana showed one in five children (20%) for the five-year period preceding the survey.

### 3.2. Complementary Log-Log Regression Model on Sociodemographic Determinants of Malaria

To examine the background variables that determine malaria cases among children under the age of five, four sequential multivariate models of complementary log-log were constructed (Table 2). In the four models, only three out of the selected sociodemographic variables had significant relationship with malaria among under-five children. These include region of residence, age of child, and ownership of mosquito net in the household.

In both models, 3 and 4, malaria in children showed a significant relationship with the Central Region, Volta Region, Ashanti Region, Brong Ahafo Region, the Northern Region, and the Upper East and West Regions except the Greater Accra and the Eastern Regions. Thus, in the final model, under-five children from the Central Region (OR = 3.129, *P* < 0.001), Volta Region (OR = 2.218, *P* < 0.05), Ashanti Region (OR = 2.550, *P* < 0.001), Brong Ahafo Region (OR = 2.992, *P* < 0.001), the Northern Region (OR = 2.412, *P* < 0.01), and the Upper East (OR = 2.325, *P* < 0.01) and West Regions (OR = 2.080, *P* < 0.05) were more likely to have malaria cases compared to their counterparts from the Western Region.

Child's age was also one of the factors that had significant relationship with malaria among under-five children. The odds of malaria were quite higher among children aged 12–23 months (OR = 2.514, *P* < 0.001), 24–35 months (OR = 2.958, *P* < 0.001), 36–47 months (OR = 3.627, *P* < 0.01), and 48–59 months (OR = 3.480, *P* < 0.05) compared to their counterparts who were aged less than 12 months. In terms of ownership of mosquito net, children from households which owned mosquito nets were 0.694 (*P* < 0.05) times less likely to contract malaria compared to their counterparts from households which owned no mosquito net. However, factors including mother's level of education, religious affiliation, ethnicity, wealth status of household, mother's marital status, mother's age, type of residence, and sex of child had no significant relationship with malaria prevalence among under-five children in Ghana for the five-year period preceding the survey.

## 4. Discussion

This paper sought to examine the factors that determine or predict malaria cases among under-five children in Ghana using a nationally representative secondary data drawn from the 2008 Ghana Demographic and Health Survey. The study revealed that malaria cases were fewer among children less than one year. This may be as a result of the fact that children below one year are protected from malaria because of the antibodies they acquire from their mothers during pregnancy [13]. Male children also experienced more malaria cases than their female counterparts. This is consistent with the finding of a study in Nigeria where malaria was found to be higher among male children under five years than their female counterparts [14], perhaps, because male children may be biologically susceptible to infectious diseases compared to their female counterparts. Malaria cases were also found to be more prevalent among children of older mothers. This could be as a result of the fact that children of older mothers may be weaker in immunity due to maternal depletion resulting from higher number of births that older mothers may have compared to the younger mothers. This is quite contrary to findings from a study in Kenya by Magadi [15] that morbidity rates of malaria were higher in children born to younger mothers aged 15–19 than older mothers.

Furthermore, the study revealed that more children from the rural areas had malaria cases than children from the urban areas. In a similar study in Nigeria, Uzochukwu et al. [16] also revealed that mothers in rural areas reported more malaria in children than their counterparts from the urban areas. Besides, Fernando et al. [17] contend that malaria is a disease of the rural people, particularly the rural poor. This implies that rural poverty may be a reason for the higher malaria cases among the rural under-five children compared to their urban counterparts. Children of mothers from the Brong Ahafo Region, mothers with no religious affiliation, and children of Mole-Dagbani mothers also had comparatively higher malaria cases than their counterparts.

Stable marriages have also been identified to provide children with the best physical health outcomes [18]. This study confirms this assertion by revealing more malaria cases among children of divorced mothers than children of married mothers. This may be because children of divorced mothers may not have the complete care that they needed in order to survive. The results also show higher prevalence of malaria among children whose mothers had lower education. Similarly, Mahfouz et al. [19] observed that the higher the level of mother's level of education, the lower the infant and child morbidity and mortality. Thus, higher mother's education served as a protective factor against infectious diseases such as malaria. The results of the study also show that malaria cases were more prevalent among children from poor households than those from rich households. Worrall et al. [20] in a study in Nigeria also found that malaria was highest among children from homes with low income. Besides, malaria in Africa has been described as a disease of rural population and communities which are homes of the poorest of the poor [21]. That is to say, malaria cases among under-five children increase with the decrease in household income level.

With regard to the determinants of malaria cases, region of residence was found to have significant effect on malaria cases with children from the majority of the regions being more likely to contract malaria compared to children from the Western Region. The likelihood of malaria cases was found to be higher among children aged one and beyond. This higher likelihood of malaria observed among children aged 12 to 59 months could be as a result of nutrient deficiency in their diet which may render them vulnerable to malaria than those below 12 months who were still likely to be exclusively breast-fed. This is because it has been observed that micronutrient deficiencies among infants could impinge on their immune system, increasing their risk for malaria [22].

The use of mosquito net has also been found in this study as a protective factor against malaria cases among under-five children in Ghana. In another study in Nigeria, Yusuf et al. [23] found that households which reported having mosquito bed net had less malaria cases among children than those without mosquito bed net. Evidence from Tanzania also revealed that children from households which owned mosquito nets and insecticide treated nets were protected from the malaria parasite compared to their counterparts from households without mosquito nets [24]. This study therefore confirms the fact that bed net ownership and use was a protective factor against malaria among under-five children [25].

## 5. Conclusion

Region of residence, age of child, and ownership of mosquito net were the key determinants or predictors of malaria cases among under-five children in Ghana for the survey period. Ghana National Malaria Control Programme with the aid of the government should conduct more malaria prevalence surveys in the various regions to identify the malaria hot spots so that malaria intervention programmes can be targeted in order to reduce any new cases. Mothers should also be entreated to provide exclusive breastfeeding for infants and provide good nutrition for older children in order to boost their immunity against infectious diseases including malaria. The distribution and use of insecticide treated mosquito nets should also be encouraged and intensified.

## Figures and Tables

**Table 1:** Proportions of malaria among under-five children.

Variable	Number of children	Proportion with malaria
Age of child (in months)		
>12	618	12.0
12–23	551	26.1
24–35	494	24.3
36–47	505	23.4
48–59	557	15.8
Sex of child		
Male	1406	20.9
Female	1319	18.9
Age of mother		
15–19	110	18.2
20–29	1301	18.7
30–39	1020	21.1
40–49	291	23.8
Place of residence		
Urban	1038	19.0
Rural	1688	20.6
Region of residence		
Western	256	10.5
Central	265	23.4
Greater Accra	329	12.5
Volta	237	18.6
Eastern	240	15.8
Ashanti	510	25.1
Brong Ahafo	260	27.3
Northern	413	21.3
Upper East	141	22.0
Upper West	72	20.8
Ethnicity		
Akan	1246	20.2
Ga-Dangme	135	15.6
Ewe	348	15.2
Mole-Dagbani	562	21.9
Others	434	21.7
Religious affiliation		
Christian	1921	19.7
Muslim	510	22.7
Traditionalist/spiritualist	170	11.8
No religion	116	26.7
Marital status of mother		
Never married	139	17.3
Married	2439	19.7
Divorced	149	29.7
Mother's level of education		
No education	886	19.5
Primary	669	22.7
Secondary	1099	18.8
Higher	69	17.4
Wealth status		
Poor	1301	22.4
Middle	504	22.0
Rich	920	16.9

Total	2725	20.9

Source: computed from GDHS, 2008.

**Table 2:** Complementary log-log regression model on sociodemographic determinants of malaria.

Variables	Model 1	Model 2	Model 3	Model 4
Mother's level of education				
No education (ref.)	1.00	1.00	1.00	1.00
Primary	1.197	1.203	1.212	1.279
Secondary	1.092	1.114	1.081	1.152
Higher	0.907	1.147	1.116	1.293
Religious affiliation				
Christian (ref.)	1.00	1.00	1.00	1.00
Muslim	1.241	1.263	1.227	1.341
Traditionalist/spiritualist	1.060	1.070	1.043	1.080
Ethnicity				
Akan (ref.)	1.00	1.00	1.00	1.00
Ga-Dangme	0.717	0.737	1.028	1.037
Ewe	0.654^*^	0.652^*^	0.773	0.782
Mole-Dagbani	1.075	1.091	1.100	1.163
Others	1.044	1.076	1.096	1.114
Mother's marital status				
Never married (ref.)	1.00	1.00	1.00	1.00
Married	1.480^*^	1.431	1.453	1.429
Wealth status				
Poor (ref.)		1.00	1.00	1.00
Middle		1.126	1.161	1.240
Rich		0.672	0.827	0.914
Region of residence				
Western (ref.)			1.00	1.00
Central			2.865^***^	3.129^***^
Greater Accra			1.338	1.451
Volta			2.049^*^	2.218^*^
Eastern			1.639	1.712
Ashanti			2.529^***^	2.992^***^
Brong Ahafo			2.922^***^	2.992^***^
Northern			2.371^**^	2.412^**^
Upper East			2.262^**^	2.325^**^
Upper West			1.993^*^	2.080^*^
Type of place of residence				
Urban (ref.)			1.00	1.00
Rural			1.015	1.115
Age of mother				
15–19 (ref.)				1.00
20–29				0.929
30–39				0.976
40–49				0.993
Age of child				
>12 (ref.)				1.00
12–23				2.514^***^
24–35				2.958^***^
36–47				3.627^**^
48–59				3.480^*^
Sex of child				
Male (ref.)				1.00
Female				0.915
Ownership of mosquito net				
No (ref.)				1.00
Yes				0.694^*^

Source: computed from GDHS, 2008; ref.: reference category; ^*^ *P* < 0.05, ^**^ *P* < 0.01, and ^***^ *P* < 0.001.
